# African rainforests: past, present and future

**DOI:** 10.1098/rstb.2012.0312

**Published:** 2013-09-05

**Authors:** Yadvinder Malhi, Stephen Adu-Bredu, Rebecca A. Asare, Simon L. Lewis, Philippe Mayaux

**Affiliations:** 1 Environmental Change Institute, School of Geography and the Environment, University of Oxford, Oxford, UK; 2 Forestry Research Institute of Ghana, UPO 63, KNUST, Kumasi, Ghana; 3 Nature Conservation Research Centre, Accra, Ghana; 4 Forest Trends, Accra, Ghana; 5 Department of Geography, University College London, London, UK; 6 School of Geography, University of Leeds, Leeds, UK; 7 Institute for Environment and Sustainability, Joint Research Centre, European Commission, Ispra, Italy

**Keywords:** Africa, tropical rainforest, climate change, deforestation, hunting, logging

## Abstract

The rainforests are the great green heart of Africa, and present a unique combination of ecological, climatic and human interactions. In this synthesis paper, we review the past and present state processes of change in African rainforests, and explore the challenges and opportunities for maintaining a viable future for these biomes. We draw in particular on the insights and new analyses emerging from the Theme Issue on ‘African rainforests: past, present and future’ of *Philosophical Transactions of the Royal Society B*. A combination of features characterize the African rainforest biome, including a history of climate variation; forest expansion and retreat; a long history of human interaction with the biome; a relatively low plant species diversity but large tree biomass; a historically exceptionally high animal biomass that is now being severely hunted down; the dominance of selective logging; small-scale farming and bushmeat hunting as the major forms of direct human pressure; and, in Central Africa, the particular context of mineral- and oil-driven economies that have resulted in unusually low rates of deforestation and agricultural activity. We conclude by discussing how this combination of factors influences the prospects for African forests in the twenty-first century.

## 1. Introduction

In recent decades, there has been a surge of interest in tropical forests, as there is increased appreciation of the rich biodiversity they host and the many roles they play in the functioning of the Earth system at local, regional and global scales. Of the world's major tropical forest regions, most research and policy attention has focused on the Amazon region, the world's largest tropical forest bloc, and to a lesser extent on Southeast Asia, the third largest tropical forest region. By contrast, the world's second largest tropical forest region, the tropical forests of Central and West Africa (termed the Guineo-Congolian region) have been relatively neglected. This has been for a number of reasons, including challenging and fragmented politics, civil conflicts and logistical as well as infrastructure challenges. Nevertheless, there is an extensive amount of research activity in the African rainforest zone that has rarely been compiled in a single interdisciplinary volume.

This review paper synthesizes the insights emerging from the theme issue on ‘African rainforests: past, present and future’ of *Philosophical Transactions of the Royal Society* [1]. This issue has explored African humid forests from a variety of perspectives, including archaeology, palaeoecology, ecology, climate science, satellite remote sensing, global climate-vegetation modelling, international policy and social science. All tropical continents and regions are different in their climate, ecology, human context and contemporary pressures. This special issue presents a synthesis of knowledge (including several commissioned reviews) and cutting-edge research from the least understood of the major tropical forest regions. It highlights the many unique features of the African forest biome.

First, it is necessary to acknowledge the limits of this theme issue. It focuses on the humid tropical forest biome (the ‘rainforests’). There are many other valuable biomes in Africa, most notably the extensive dry open forest, savanna and grassland biomes, and also mangroves, afro-montane ecosystems and others. All of these are valuable and fascinating ecosystems, which for reasons of brevity are not covered in this volume. Second, many of the analyses presented dwell on the largest biogeographic unit that accounts for 95% of African rainforests, the Guineo-Congolian forests of West and Central Africa. We particularly focus on Central Africa (technically the Congo–Ogooué Basin and contiguous forests, hereafter termed the Congo Basin for brevity), which accounts for 89% of African rainforests. The submontane forest patches of East Africa and the unique forests of Madagascar receive less detailed attention here. However, a number of studies, including those on deforestation [2,3], woody encroachment [4], climate [5] and forest structure and biomass [6], do extend beyond the Guineo-Congolian forest zone.

## 2. The extent, biomass and structure of African rainforests

The issue presents two new, ground-breaking analyses of the extent, biomass and structure of the African rainforest realm. Mayaux *et al.* [2] present a new wall-to-wall map of humid forest cover in Africa, for the year 2005, at 250 m resolution, dividing the region into four classes: lowland rainforest, swamp forest, rural complex (10–30% tree cover and more than 50% croplands) and other land cover (remaining savanna, croplands, etc.). The analysis makes key an advance over previous maps by using the twice-daily passes of the Terra and Aqua satellites, with their on-board moderate resolution imaging spectrometer. The frequency of the satellites' overpasses enables sufficient collection of cloud-free data, even over the cloudiest regions.

They estimate that the total lowland humid and swamp forest area for Africa is 1 998 290 km^2^, of which 89.3% is in Central Africa, 6.0% in West Africa, 2.2% in Madagascar and 2.4% in Eastern Africa. In terms of countries, the Democratic Republic of Congo (DRC) is the forest giant, accounting for 53.6% of Africa's lowland rainforest area, followed by Gabon (11.2%), the Republic of Congo (10.4%) and Cameroon (10.0%). The remaining countries account for 14.8% of total lowland rainforest area.

Compared with the American and Asian tropics, there have been very few systematic regional studies of even the basic attributes of African forests such as biomass, species diversity and structure. Lewis *et al.* [6] present the first large-scale analysis of how the biomass and structure of old-growth African forests vary across the region. Presenting data from 260 forest plots, they find that African forests have a mean above-ground biomass of 395.7±14.3 Mg dry biomass ha^−1^, and the mean increases to 429 Mg dry biomass ha^−1^ in Central Africa. This is much higher than the mean value of 289 Mg ha^−1^ reported for Amazonia, and comparable with the mean 445 Mg ha^−1^ reported for the famously high biomass forests of Borneo. Another key feature is that African forests have a lower mean stem density (426 ± 11 stems of 100 mm diameter or more, compared with around 600 in Amazonia or Borneo), consisting of proportionately many more larger-sized trees than other continents, and fewer small trees. The reasons for these differences are mysterious. Does this imply much lower disturbance rates in Africa that favour longer-lived trees, or perhaps higher net primary productivity? Does the (until recently) pervasive presence of forest elephants, gorillas and other megafauna reduce the number of small stems and increase nutrient availability [7], and do these factors also explain the difference between higher biomass Central Africa and lower biomass West Africa? Does some aspect of the physical environment promote high biomass, for example, via lower rainfall, possibly sunnier conditions or deep weathered soils? Another striking feature is that on average the plots seem to be increasing in biomass over time [8,9], which may be a response to global atmospheric change, or else a legacy of ongoing recovery from past human or climatic disturbance or a combination of the two. These agents of change are discussed further below.

## 3. A history of disturbance

Another key feature of African rainforests is the heavy influence of past disturbance, both through climate change and human activity. The rainfall regime of much of humid Africa is close to the lower threshold of rainforest viability [10,11]; hence, small changes in rainfall or in intensity or duration of the dry season can cause fairly large-scale changes in rainforest or savanna cover.

Willis *et al.* [12] summarize the history of climate change in Africa since the Last Glacial Maximum, 12 000 years ago. The theory that tropical rainforests retreated into small refugia during the cold arid phases of the Ice Age was first proposed for Amazonia [13] but has largely fallen out of favour for that rainforest region [14] with evidence that continuous, albeit drier, lowland forest persisted across Amazonia throughout the Ice Age. In Africa, by contrast, there is strong evidence of rainforest retreat during dry periods [15] that leaves a legacy in the current distribution of African vegetation species, with slow-dispersed species (e.g. those that are dispersed ballistically rather than by animals or wind) still expanding slowly out of refugia [16]. Much of the present-day rainforest belt in West and Central Africa would have been savanna–grasslands in the Ice Age, and the rapid climate fluctuations would have favoured species that dispersed and colonized rapidly. The low plant diversity of most African rainforests compared with Amazonian or Asian counterparts under similar modern climates may be a legacy of this history of frequent climate and vegetation change [17].

After the Ice Age, much of Africa experienced a rapid transition to a climate warmer and wetter than present, and these conditions broadly persisted over the period 11 000−4000 years BP, an interval referred to as the African humid period [12]. The higher rainfall and deeper penetration of the West African monsoon in this period was associated with solar orbital forcing, with more intense heating of the Sahara over the northern summer driving a stronger monsoon. At the peak of the African humid period (11 000−8000 years BP), the African vegetation zones extended much further (up to 400–500 km) to the north of their present position, and the Sahara was criss-crossed by lakes, rivers and inland deltas [12,18].

As solar orbiting forcing gradually shifted, the climate in much of West and Central Africa shifted to a drier regime around 4000–2000 years BP [12]. This period is associated with retreat of forest cover and expansion of savannas, but also expansion of human activity. Oslisly *et al.* [19] present a comprehensive synthesis of human presence in Atlantic Central Africa. There is some debate about the extent to which human activity caused forest retreat, or simply took advantage of the opening up of the forest margins in a drier climate [20–23]. Both factors are probably important, but the timing of forest retreat favours climate change as the underlying driver of forest retreat.

Oslisly *et al.* [19] paint a compelling picture of waves of human settlement, punctured by periods of population collapse. The Late Stone Age began in Central and West Africa around 40 000 years BP, and persisted until around 3500 years BP, when stone-working hunter–gatherers gave way to Neolithic farmers migrating into the region from the Sahel. These early farmers practiced rudimentary slash-and-burn, and took advantage of climate drying and forest fragmentation to penetrate into the forest block. Soon after (approx. 2800–2500 BP), a wave of Iron Age farming spread south. These farmers increased rapidly in numbers and likely had a profound effect on the forest: with their iron tools, they had the potential to slash-and-burn more extensively in the forest, and the process of iron smelting also required great quantities of charcoal. They also probably increased fire frequencies in savannas, favouring the spread of savannas into forests. Iron Age settlement peaked around 1900 years BP, but was followed around 1600–1000 BP by an extensive population crash, suggesting that Atlantic Central Africa was almost devoid of people at this time. The cause of this crash is still a mystery, but disease has been suggested, with sleeping sickness or Ebola as possible culprits. As the human population crashed, the forest likely expanded and recovered.

From 1000 years BP, a second wave of metalworkers settled into the forest region. This new expansion peaked around 500 BP, but then suffered a new crash. This time the Atlantic slave trade may be the likely cause of collapse, both by direct removal of people and by causing abandonment of poorly defended agricultural areas. The subsequent colonial policy (in some countries) of forced resettlement of rural communities along transport routes also contributed to reduced agricultural activity across broad expanses of forest. Again, the forest biome probably expanded and increased in biomass. Many of the forest tree species that characterize African forests from West Africa to Congo are characteristic advanced succession species (such as the *Aucoumea klaineana* (okoumé) that dominates parts of Gabon), indicating a forest age of a few hundred years. Another example, Gond *et al.* [24] used a satellite-based analysis of seasonality to describe a broad band of low diversity, disturbed and semi-deciduous forest in the Sangha River Interval, and area of sandier soils and possible lower rainfall that divides the western Congo Basin from the eastern Basin. In this presently low population density region, there is abundant evidence of past cultivation, and past retreat of the forest [19].

Hence, the story of Africa's forests has been one of climatic change even throughout the Holocene, and altering levels of direct human impacts. The ecology and biodiversity of Africa reflect this history of disturbance, with lower levels of wet-affiliated species than expected for the current climate [17], and a high abundance of large trees that disperse and grow rapidly and perhaps represent an advanced stage of succession (ecological ‘scar tissue’ spreading over a disturbance [25]). These species may therefore be more adaptable to contemporary climate change and disturbance, but this hypothesis remains to be rigorously tested.

The African forests have been through phases of dense human settlement, and also population collapse, as in Central Africa 1600–1000 BP and more widely since 500 BP with the Atlantic slave trade and forced resettlement. Currently, there is very high population pressure in some regions (e.g. across West Africa, and the northern and eastern margins of the Congo Basin), but low population pressure in the central and western regions of the basin. The human populations of the American tropics also witnessed a massive (disease-associated) collapse after European colonization of the Americas. However, pre-collapse human impacts in much of Amazonia away from drier fringes and river margins are likely to have been less [26,27] despite some arguments to the contrary [28]. In particular, the Americas never experienced an Iron Age, which in Africa produced more effective tools for clearing forest and greater demand of wood fuel for smelters. The climate of African rainforests is also, on average, much drier than most of the tropics, and therefore more suitable for a greater variety of successful agricultural crops.

## 4. Deforestation: patterns and causes

Deforestation is the most visible and prominent agent of contemporary change in tropical forests. Globally, rates of deforestation at the start of this century were around 5.4 million ha yr^−1^ [29], contributing about 1.30±0.24 Pg C of emissions to the atmosphere, about 15±3% of global human-caused CO_2_ emissions [30]. The rates of deforestation in Amazonia and Southeast Asia are fairly well defined [29,31], because national reporting capacity in countries such as Brazil and Malaysia is high, and in area terms much of it is driven by medium-to-large-scale clearing for cattle ranching and soya bean (in Amazonia) and oil palm plantations (Southeast Asia), both of which are fairly easy to detect by satellite. In Africa, there has been much more uncertainty about rates and patterns of deforestation, because national reporting capacity has been low, and the mode of deforestation is primarily small-scale clearing by subsistence farmers. This requires high-resolution imagery such as those provided by Landsat.

Mayaux *et al.* [2] present a new analysis of deforestation across the African rainforest zone over two 10-year intervals 1990–2000 and 2000–2010, taking advantage of the recent free availability of the Landsat archive to conduct high-resolution time-series analyses of 10 × 10 km areas distributed on a regular grid at each integer latitude and longitude intersect. They estimate that African rainforest net deforestation rates were 0.59 million ha yr^−1^ between 1990 and 2000, and decreased to 0.29 million ha yr^−1^ over 2000–2010. This rate is four times smaller in absolute terms than that in Latin America, and the proportional rate is also smaller (0.3% versus 0.4%; [32]). The absolute rates in Asia are also three to four times larger [31]. Hence, from a global perspective, much of Africa is still a relatively low rainforest deforestation continent, contributing only about 11% to global gross deforestation. This reflects the almost complete absence of agro-industrial scale clearing in Africa, which accounts for around 55% of global tropical rainforest deforestation in the 2000–2005 period [29]. At a finer scale, there are hotspots of deforestation in West Africa and the fringes of the Congo Basin, and no evidence of a decline in deforestation in Madagascar. Rates of deforestation seem to rise when rural population densities rise above around 10 people km^−2^, or the area of cropland rises above 10%, in a 10 × 10 km^2^ square. Hotspots of deforestation are also found around transport networks and close to cities, including areas suitable for agriculture within 5 h travel time to major markets, and wood fuel and charcoal provisioning within 12 h from a city. These are very similar to the ‘waves of degradation’ documented in East Africa, which conform to economic models based on the value of products derived from tropical landscapes [33].

The pattern of low and declining rates of deforestation in African rainforests (and particularly in Central Africa) is somewhat surprising and warrants further exploration. Rudel [3] presents a national-scale analysis of the drivers of deforestation. He notes several key features of human ecology that are important in the context of Africa: the population has long been much poorer and more rural than that in Latin America but now has the highest rates of urban population growth (3.7% per year between 2000 and 2005), much of the urbanization is occurring without industrialization (e.g. domestic energy supply is mainly wood fuel), and almost all of the significant Congo Basin countries that account for almost 90% of Africa's rainforest cover have large extractive oil and mineral industries (with Cameroon being a notable exception). This extraction of oil and minerals triggers economic booms that set off cascades of economic side effects (such as high labour costs, less competitive agricultural exports, more food imports) that retard agricultural expansion, accelerate urbanization and slow rates of deforestation in remote, rural regions. Deforestation is particularly concentrated in peri-urban areas and along transport routes [2], to meet the demands of the concentrated and growing urban population. Consistent with this hypothesis, both Rudel and Mayaux *et al.* [2] find that the lowest rates of deforestation tend occur in mineral- and oil-rich nations. It is worthwhile to consider the particular circumstances of the rainforest giant, the DRC, separately in this analysis, as some different dynamics may be occurring there. It accounts for over 50% of African rainforest area [2], has high oil and mineral receipts (around 90% of exports) and yet low rural income and has experienced high levels of political instability that is still pervasive in some regions. Albeit from a very low baseline rate, it is experiencing increasing levels of deforestation [34], in particular around its rapidly urbanizing zones.

## 5. Selective logging

Logging is a more cryptic agent of change, challenging to monitor by satellite. In popular usage, logging is often confused with deforestation, partially, because, in temperate regions, logging is often associated with clear-cutting of low diversity forests. In the high diversity African tropics, there are typically only one to two timber trees harvested per hectare [35], and it makes little economic sense to clear cut forests remote from markets. This contrasts with Southeast Asia, where the dipterocarp-rich forests yield many more timber species, and are much more intensively logged (around 10–20 trees per hectare) and heavily damaged by logging. In principle, a tropical forest can be logged with low impact, and given sufficient recovery time before return logging, this could be sustainable, although the definition of what is sustainable logging is much debated [36,37]. Much of the damage associated with logging comes from collateral damage to other trees, and through the opening up of logging tracks and platforms. Research from Amazonia [38] has also suggested that logging can often be the precursor of complete deforestation, and also opens up the forest to a drier microclimate and increased fire vulnerability.

In Central Africa, much of the lowland rainforest area is apportioned to long-term logging concessions, about 44 million ha [39]. Over the past two decades, there has been a noteworthy shift from unsustainable ‘mining’ of timber resources to at least an aspiration for sustainable management and conservation of timber resources. Between 1990 and 2002, most countries in Central Africa redefined their forestry laws to make management plans for logging concessions mandatory. Over 18.6 million hectare of forests in Central Africa now have a management plan [40].

Gourlet-Fleury *et al.* [35] present results from a long-running (24 year) logging experiment in the Central African Republic to assess the effects of disturbance linked to logging and thinning on the structure and dynamics of the forest. They find that the forest responded fast to the logging disturbance, rebounding back to near original levels of biomass after 24 years, but the volume of timber did not recover so quickly. With the logging of one to two trees per hectare removed every 30 years, the forest appears to maintain biomass and function. However, the most highly valued timber species take much longer to recover, so in terms of maintenance of timber species such logging is still effectively ‘mining’ of timber resources.

Mayaux *et al.* [2] and Rudel [3] explore the possible linkages between logging and subsequent deforestation. They find little evidence of logging leading to deforestation, either at national or local scales. One key difference from Amazonia is the lack of an active colonization frontier with pressure to clear logged forest. The loose network of logging tracks, owing to the light exploitation density, combined with the low population density, does not provoke the critical conditions for deforestation, except around a few concessions in the DRC. They do however greatly facilitate hunting pressure, which can have knock-on consequences on forest structure and biomass and discussed below.

## 6. Woody encroachment

Many analyses of forest cover change are biased towards detecting and quantifying forest loss. Much less attention has been focused on areas where forest cover seems to be increasing. Such increases in forest cover (or woody encroachment) may be caused by locally reduced direct human pressure (e.g. where rural depopulation is underway), particularly changes in the fire regime, by increasing rainfall (as suggested by most climate models for much of eastern Africa: [5,41]) or by rising atmospheric CO_2_ concentrations favouring tree growth over grasses, and enabling tree saplings to better escape grass-associated ‘fire traps’ in mixed tree–grass regions [42].

Mitchard & Flintrop [4] present an analysis of this phenomenon of woody encroachment, by both conducting a systematic review of published instances of woody encroachment in Africa, and by conducting a long-term (1982–2006) study of satellite-derived normalized difference vegetation index (NDVI). This is a vegetation index that is a measure of greenness, across the mixed tree–grass systems of Africa. In the peak dry season when grasses are yellow, NDVI is likely to be a good proxy for tree cover. They find evidence of increased dry season NDVI across the north fringes of the rainforest biome, but general loss of greenness in the southern African Miombo woodland regions where there is heavy degradation pressure. All documented studies of woody encroachment coincide with an increasing trend of NDVI at the same locale (though not always significant).

In South Africa, there is substantial evidence that the woody encroachment is associated with increased CO_2_ rather than with rainfall or with reduced land use pressure [42]. It is plausible that a similar factor is driving the encroachment in more northern regions, although recent increases in precipitation following drying in the 1970s and 1980s may also be a factor [43]. Lewis *et al.* [8] have noted that intact old-growth forests in Africa have been increasing in biomass, possible in response to higher CO_2,_ something also noted by Gourlet-Fleury *et al.* [35] in the control plots in their logging experiment. Hence, the consistent phenomenon of CO_2_ fertilization may be increasing forest biomass in biomes ranging from wet rainforests to dry tree–grass systems. Thus, calculations of the carbon sink across the tropical forest biome should include that from woody encroachment, not yet well estimated, in addition to that already estimated for rainforests.

## 7. Defaunation

Other aspects of human-induced change in rainforests are less visible to satellites than deforestation, but more pervasive. Among the most important of these is hunting, whether for bushmeat or for international trade in wildlife parts such as ivory and skins. Abernethy *et al.* [44] present a review of the factors driving hunting in Central Africa, and a synthesis of data on the direct impacts of hunting on the functioning of forest ecosystems.

Africa is the origin continent of humanity, and African rainforest species have evolved in the presence of hominid hunters and their ancestors. This is in sharp contrast to, for example, the Americas, Madagascar and Australia, which suffered a major loss of megafauna around the time of arrival of the first human hunters [45]. As a result, until decades to centuries ago, African rainforests have held abundant populations of megafauna such as elephants and large primates. However, modern hunting pressure, driven by increased commercial trade, has resulted in depopulation and local extinction of many larger rainforest mammals. The remaining forest reserves of West Africa, often structurally intact but devoid of medium and large fauna, are ‘empty forests’. Now, the most striking rates of defaunation are in Central Africa. A massive increase in ivory poaching has led to 62% of Central Africa's forest elephants being lost within a decade (2002–2011), with no sign of a decline in the rate of poaching [46]. Large declines are also occurring in ape populations, which declined by 50% in a study across Gabon over 1984–2000 [47]. At a less dramatic but even more widespread scale, bushmeat hunting affects around 178 species in Central Africa, with over half these species deemed threatened by this hunting [44]. It has been estimated that over 6.5 million tonnes of bushmeat per annum are extracted from tropical forests, rising by over 100 000 tonnes each year, of which 90% is extracted from African forests [48,49].

These massive rates of defaunation represent a direct loss of some of the most unique and valuable aspects of African forests. They also have knock-on effects, such as loss of top predators such as leopards as their prey become rare, effects on forest structure, seed dispersal and recruitment [50] and possible changes in nutrient diffusion through the forest [7]. These can lead to shifts in the tree species composition of the forest [51]. The loss of high wood density animal-dispersed species may also lead to a reduction of biomass in the forest [44]. By contrast, Lewis *et al.* [6] make the suggestion that by ‘culling’ small trees, megafauna might actually increase the preponderance and lifetime of larger trees and the overall biomass of rainforests, which may explain why African rainforests have higher biomass than Amazonia, where the megafauna went extinct around 10 000 years ago. This might partially explain why Central African rainforests have higher biomass than West African ones, and why Africa has higher biomass than Amazonia, where the megafauna went extinct around 10 000 years ago. Consistent with this, a recent report tracking forest responses, which a forest was hunted to silence, showed a dramatic increase in tree stem density [52]. The ongoing expansion of logging trails into the remotest parts of the African rainforest realm is likely to only intensify hunting pressure and its consequences, as accessible areas have much higher bushmeat extraction than inaccessible areas [44].

## 8. Climate and climate change

A number of papers in this theme issue explore the theme of climate change, a looming but poorly quantified and understood threat to the African rainforest realm. James *et al.* [5] and also Zelazowski *et al*. [11] conduct an in-depth analysis of multiple climate-change projections for the region. All climate models agree that the African rainforest regions will warm, with a mean rate across models of 0.8–1.0°C per 1°C of global warming. Hence, the African rainforest realm is likely to warm by 3–4°C over this century under the most likely emissions scenarios. How tropical organisms will adapt to these this warming remains unclear—some studies argue that tropical organisms are particularly sensitive to warming because of their limited seasonality and interannual variability in temperature [53,54]. Similarly, in the lowland tropics organisms are required to travel long distance to maintain a constant temperature as mean air temperature increases [55]. Conversely, some species more than three million years old may have experiences warmer temperatures than those expected in the coming decades and may therefore be able to adapt [56].

For rainfall, a simple average of change in total annual rainfall across models suggests little overall change in the central and western regions of equatorial Africa, and wetting of the eastern regions. Such an analysis would be deeply misleading, however. When metrics of the seasonality of rainfall are considered, most models predict intensified dry seasons in the western Congo Basin. Outside of the West and Central African core, most models suggest that East African forests will get wetter, but Madagascar's forests will experience increasingly severe water stress. The length of and intensity of the dry season is likely to matter more than the mean rainfall in determining the viability of rainforests [11]. On the other hand, a number of models still suggest wetting of western equatorial Africa, hence averaged across models there is little overall trend. James *et al.* [5] delve further into the patterns and mechanisms that distinguish the ‘dry’ models from the ‘wet’ models. They show that drying is associated with enhanced sea surface warming over the Indian Ocean, together with warming in the tropical North Atlantic causing a northward shift in the inter-tropical convergence zone. Warming in both these regions drives enhanced uplift of air in these regions, which in turn drives intense subsidence in western equatorial Africa. The review identifies these ocean-driven atmospheric circulation shifts as the likely atmospheric mechanism to the potential threat to the rainforests of the western Congo Basin, and it is a clear research priority to identify whether the dry models (the majority) or the wet models are more plausible. There is clear evidence that Congo Basin and West African forests have retreated substantially because of climate change even as recently as 3000 years ago [19,20], suggesting that the climate change-induced forest retreat is certainly feasible.

Probably the greatest challenge in understanding and projecting the future climate of the African forest realm, and in particular the Congo Basin, is that the current climate is so poorly observed and understood. This deficiency is of global significance, because the Congo Basin is the second most important convective ‘engine’ of the global atmospheric circulation after the Maritime Continent (insular Southeast Asia and the surrounding waters) and is also the region of highest lightning strike frequencies on the planet [57]. In transition seasons (March–May and September–November), the Congo Basin dominates global tropical rainfall [58]. Despite this importance, the Congo Basin is poorly studied because of the dearth of available ground-based climate observations from the region, particularly in the past few decades [58]. The number of rain gauges reporting from this vast region declined from more than 50 in the period 1950–1980, to less than 10 over the period 1990–2010 [58]. Much of this decline is driven by political instability: for example, only three meteorological stations from the DRC reported to the Global Telecommunication System in 2013, despite this country accounting for over half of Africa's rainforests. Washington *et al.* [58] analyse a range of model and satellite observation products for the region, and show there is little agreement in estimates of the distribution and total quantity of rainfall in the region (e.g. whether the western or eastern Congo Basin is wetter). The datasets differ by a factor of at least two, and in absolute terms by at least 2000 mm per year.

Clearly, there is a need to re-establish a ground meteorological observation system over the region. In the short term, however, Washington *et al.* [58] suggest that even a short-period, intensive observation campaign that targets the atmospheric circulation and water transport could yield vital insights and clues to the true rainfall regime of the region. Such a campaign would combine radiosondes, weather radar and aircraft data collection, as done for West Africa past decade by the African monsoon multidisciplinary analyses (AMMA) programme [59]. There is also evidence that the tropical African climate is more predictable in the short term (seasonal and interannual scale) than many other regions because of its strong links to Atlantic and Indian Ocean sea surface temperatures [60].

Beyond changes in a mean state of the climate, increasing variability and seasonality are also key indicators of a changing and unstable climate system. An important signature is the intensity and frequency of extreme drought events. Asefi-Najafabady & Saatchi [61] analyse time series of precipitation in Central and West Africa using time series of ground interpolated observations, and, since 2001, tropical rainfall monitoring mission satellite rainfall estimates. They show substantial interannual variation in dry season intensity (particularly in 2005, 2006 and 2007) as part of a general drying trend in West Africa and the northern Congo Basin that started in the 1970s.

But are such drought events linked to global climate or just a signature of natural variability? The drying observed in West Africa since the 1970s is thought to be largely driven by natural variability [62], but is there also a signature of global warming either in this region or across the Congo Basin? Attempts to answer such questions are termed attribution studies. They require very large ensembles of climate model simulations, so that the statistics of a rare event such as an extreme drought can be estimated some with confidence. Otto *et al.* [60] present an attribution analysis of drought events for the Congo Basin, the first attempt at an attribution study for a tropical forest region. To generate the large ensemble of climate model outputs, they use output from the weather@home project, which runs climate models distributed across volunteers' computers. They compare simulated current climate with a counterfactual current climate in the absence of greenhouse warming, to see if there is a statistically significant change in the probability of modelled drought events that could be attributed to global warming. In the case of the Congo Basin, they find no conclusive evidence that changes in drought frequency can be attributed to global warming. All such attribution studies rely on the model's ability to simulate the region's climate, and in the case of Central Africa, the lack of observational data make it very hard to be sure what the actual climate is.

If there is a mean drying, or if variability becomes more extreme, then how will African forests respond to such events? From one perspective, African forests are more vulnerable to climate change because they sit close to a rainfall threshold that favours savannas over rainforests. The previous extensive contractions of the rainforests in the Ice Age and the drier periods of the Holocene indicate that extensive climate-driven rainforest loss is clearly quite possible. On the other hand, as long as the rainfall threshold is not crossed, the disturbance-adapted nature of many African forests may favour more resilience than their Amazonian or Asian counterparts [12,19]. Willis *et al.* [12] suggest that the palaeoecological record in Africa indicates a nonlinear and spatially heterogeneous response to a change in climate: little change until some threshold is crossed, then a rapid shift in the ecosystem.

There is evidence of at least short-term resilience to drought. Fauset *et al*. [63] explored the response of Ghanaian forests to the drying event in the 1970s. They found that after 20 years of exposure to a drier climate, the composition of the forests shifted towards more drought-adapted, deciduous species, and plot-level biomass actually increased over time. This suggests a relatively rapid response in forest composition to perturbations, which gives some resilience to the persistence of the forest biome. However, in areas which burnt during a particularly severe drought year in 1983, there was a transition to non-forest habitats that persists to the present day. This highlights the importance of interannual variability, fire and human pressures in mediating the resilience of forests to climate change.

At a larger scale, Asefi-Najafabady & Saatchi [61] examined changes in canopy structure and moisture content using microwave satellite data. They found that, despite substantial drought events over the past decade, there was little evidence of lingering effects on the forest canopy. This contrasts with a study using identical methods in Amazonia [64], which found a lingering multi-year response in the canopy of southwest Amazonia. Amazonia has had a more stable climate history than tropical Africa, and may have higher abundance of species more accustomed to a stable climate, and hence more vulnerable to climate change.

A key factor to consider is the rapid rise of atmospheric CO_2_, by the end of this century to levels probably not experienced for over 50 myr. This is a feature of twenty-first century change that makes it very different from previous changes during the Ice Ages. High atmospheric CO_2_ can have a number of consequences: it can directly stimulate plant photosynthesis and growth, it can increase the water use efficiency of plants in dry environments, and it can increase the competitive advantage of trees over tropical grasses, enabling woody encroachment and expansion of forests into savannas. The ongoing increase in woody biomass in old-growth African rainforests reported by Lewis *et al.* [8], and Gourlet-Fleury *et al.* [35] and the woody encroachment of savannas reported by Mitchard & Flintropp [4] may both be signatures of rising CO_2_, although other factors such as recovery from disturbance cannot be completely ruled out.

Fisher *et al.* [65] present a synthesis of outputs from nine global vegetation models for the African rainforest biome. They find that most models predict a current increase in biomass in response to CO_2_ fertilization of about 0.5 Mg C ha^−1^ yr^−1^, very close the field-observed value of 0.6 Mg C ha^−1^ yr^−1^ reported by Lewis *et al.* [8]. The models suggest the rate of absorption of CO_2_ flux has been declining in West Africa and the northern Congo because of the recent dry decades. Most models predict the carbon sink to continue to decline in strength over this century: the exact timing and magnitude of that decline causes model predictions to diverge as the century progresses.

Hence, the picture emerging from field monitoring, palaeoecological work, satellite monitoring and ecophysiological modelling is one of resilience to occasional droughts, moderate warming and moderate long-term drying. This contrasts with the high rates of climate-change-related extinction produced by bioclimatic envelope models. This resilience is perhaps greater than for other major tropical forest regions because of Africa's history of climate change and human pressure: the potentially most vulnerable species are already extinct. However, the palaeoecological data also point to there being thresholds (perhaps closer in African forests than in other places), when the forest can convert rapidly to savannas. Such thresholds may be amplified when forest–climate feedbacks are considered, where the retreat of the rainforest reduces recirculation of water to the atmosphere and thereby reduces rainfall, which causes further forest retreat. Within the constraints of our limited understanding of the climate circulation of the region [58], there is evidence that the rainforest plays a critical role in recycling precipitated water back to the atmosphere [66,67], and regional climate model simulations suggest that extensive loss of forests causes not only large decreases local rainfall but also affects the African monsoon and hence rainfall patterns across many more arid, marginal areas of this generally dry continent [68].

## 9. Threats and opportunities

### (a) The threat of dramatic change

The unique demographic and economic circumstance of much of humid tropical Africa presents the opportunity to possibly raise incomes without extensive clearance. However, a major threat to the forest biome is the potential of a shift to commercial agro-plantations. If poorly planned, then these industrial croplands could lead to extensive loss of forests as witnessed in Southeast Asia, and in soya bean regions of Amazonia, especially when combined with poor governance. On the other hand, the international commercial organizations involved in operating such plantations may be more amenable to land use planning and international governance pressure. In particular, any discussion of the future of the African forest biome as a whole needs to consider the unique importance and particular circumstances of the DRC, with its combination of high mineral resources, high rates of deforestation, fragile governance and civil conflict.

Climate change is a big unknown, because we simply do not know enough about the nature of the present and future climate of tropical Africa, nor about the response of vegetation to that climate change. We know it will almost certainly be 3–4°C warmer in the forest realm by 2100 that atmospheric CO_2_ concentrations will be much higher, and that rainfall variability will probably be greater. Critically, we do not know what the likely patterns of rainfall change will be. James *et al.* [5] have identified that climate models tend to cluster towards two opposing modes of rainfall change, associated with sea surface temperature patterns in the Indian and Atlantic Oceans. The more negative mode would imply substantial drying and retreat of rainforests across Central and West Africa, as seems to have happened a mere 3000 years ago [15], although in the twenty-first century context the high levels of CO_2_ may to some extent partially offset the negative impacts of drying on forest vegetation. In the opposing ‘positive’ mode of climate change, there would be increased rainfall across Central and West Africa resulting in substantial woody encroachment in the savanna biomes.

### (b) Opportunities for rainforest conservation

There are new opportunities emerging to support the conservation of forests. The opportunity attracting the most attention is through international climate change mitigation funding for reducing emissions from deforestation and forest degradation and conservation of forest carbon stocks, sustainable management of forests and enhancement of forest carbon stocks (REDD+). That is, payments would be made based on verified reductions in carbon emissions in a country relative to an agreed emissions baseline in the absence of any mitigation activity. This represents a potentially transformative opportunity for a more sustainable future for Africa's forests, but also faces a number of challenges, foremost of which are an international agreement on climate change mitigation targets and strategies, and effective implementation of the international flow of funds. At the national and subnational level, REDD+ also brings huge challenges owing to limited national capacity to implement and monitor the complexities of land and tree tenure arrangements, and ensuring safeguards for forest peoples and for biodiversity protection. The slow pace of progress towards any international agreement on climate change is resulting in disenchantment with REDD+. Nonetheless, progress is being made in setting up national architectures and developing the capacity to implement REDD+ and monitor and report on its progress, as well as in implementing local-scale pilot projects. Maniatis *et al.* [69] examine the current flows of REDD+ finance in Congo Basin countries and the status of national engagement and capacity to implement REDD+. They find that there has already been at least US$550 million of REDD+ financing committed or disbursed, with the biggest recipients being the DRC (41%), regional entities (34%) and Cameroon (15%). There is a large disparity in terms of preparedness of REDD+, with the two largest African rainforest countries (DRC and Gabon) making substantial progress, and many of the lower rainforest area countries trailing behind. Such building of capacity and awareness of national forest resources may already be having an impact on slowing down deforestation and degradation activities in the region.

In addition to these challenges at international and national level, one key challenge for the implementation of REDD+ or other forest sustainability efforts is successful engagement by local communities in the management of their forests. There are lessons to be learnt and models to be adopted in the light of decades of experience in tropical forest conservation in Africa. Asare *et al.* [70] highlight and reviews one promising model that has emerged from Ghana: the community resource management area (CREMA). This mechanism authorizes rural communities and land users to benefit economically from their forest resources, while allowing them to manage the resources in ways that are founded upon traditional values and are compatible with local by laws and national legislation. As a mitigation strategy, the CREMA has the potential to solve many of the key local-scale challenges for REDD+ in Africa, including definition of boundaries, small-holder aggregation, free prior and informed consent, ensuring permanence, preventing leakage, clarifying land tenure and carbon rights, and enabling equitable benefits-sharing. Successful implementation of REDD+ at local scale would require African government support for CREMAs or similar mechanisms, and motivation by communities to integrate such systems within their traditional values and natural resource management systems.

We started this synthesis by pointing out that all tropical rainforest continents and regions have their unique history, climate, ecology, governance and patterns of utilization that make the prospects of their tropical forests particular to that region. This theme issue has highlighted the multifaceted uniqueness (or ‘exceptionalism’) of the African humid forest biome. Other tropical forest regions may also have some of these features in common with Africa, but this particular combination of features characterizes much of the African rainforest biome. Key among these are

— the extensive history of climate variation, biome expansion and retreat, and human interaction with the biome,
— the relatively dry and cool climate when compared with other major tropical forest regions,
— the relatively low plant species diversity and yet extremely high animal biomass (in the non-heavily hunted forests),
— complex patterns of customary and state land tenure built in long histories of low-level forest exploitation,
— the dominance of selective logging, small-scale farming and bushmeat hunting as the major forms of pressure on the rainforest biome, in contrast to the agro-industrial pressures that dominate in the tropical Americas and Asia,
— the particular context of mineral- and oil-driven economies of Central Africa, resulting in unusually low rates of deforestation and agricultural activity, and
— the particular governance and poverty challenges and civil conflict context of the African tropical forest giant, the DRC.

We conclude by highlighting some research needs that have emerged from this theme issue.

First and foremost, there is a need to build up relevant scientific capacity in African countries, building upon and supporting existing research institutions, and creating mechanisms for scholarships for study in both Africa and overseas, and for research and fellowships and infrastructure development. Sparsely funded African research institutions should have greater access to funds which can support African research priorities.

There is a need for basic ecological understanding of the African rainforest biome, which lags far behind that of the Americas and Asia. This includes understanding productivity, species distributions, drought- and temperature-sensitivity and interactions with climate and soils. This requires investment in selected intensive studies sites combined with more extensive distributed networks of study sites, both integrated and standardized with parallel efforts in other rainforest continents. Attention should also be focused on the more rare rainforest formations, such as swamp, montane, inselberg and mangrove forests, as well as the widespread wet and dry forest biomes.

If anything, our understanding of Central African climate is even weaker than our understanding of its ecology. There is a pressing need to rebuild the climate monitoring network in Africa, linking in the weather stations to global reporting networks and integrating with satellite observations. There are also opportunities for major advances in insight through a short-term, targeted campaign that focused on understanding the circulation and moisture flow patterns across Central Africa in the dry season. Similar campaigns in South America such as the Large-scale Biosphere–Atmosphere programme in Amazonia (LBA) have transformed our understanding of the Amazon region and greatly enhanced local capacity. We also need to understand the interactions between the rainforests and the atmosphere: to what extent is the rainforest a critical source of recycled water and a driver of atmospheric circulation across this generally arid continent, and how would rainfall patterns change in the event of substantial retreat of rainforests?

There is also a need to better understand and monitor the particular processes of change that predominate in Africa. What is the nature of small-scale farming and how well can it be monitored by satellites? What are the threshold levels of defaunation pressure that drive species to local extinction or functional irrelevance? How does the spreading network of logging trails affect defaunation? Can we better understand how the structure of intact African forests is affected by historical change and by ongoing defaunation? What are the interactions between forest degradation, fire risk and climate sensitivity?

In social science research, there is a need to better understand the unique interactions between urbanization, poverty, oil and mineral extraction, economic growth, wood fuel use and agriculture in an African context, and in the particular context of each African country. We need to understand the political ecology and economy at scales ranging from international through to community and individual farmer.

There is also a need to better apply the research to practical management and conservation of the rainforest biome. Of critical importance is finding platforms and processes that enable sustainable, effective management of African forests and forest resources that operate at multiple scales (local to national) and accommodate state and traditional values and norms. When large-scale agro-industry arrives in the African rainforest biome (as it has begun to do already), how can its negative impacts be minimized and development and conservation goals both be met? What are optimal strategies to protect the African fauna and the many ecosystem services that they provide? Can we reliably identify the areas of the rainforest biome most vulnerable to climate change, and manage and plan accordingly to maximize resilience of the biome, its species and its human inhabitants? Can effective mechanisms (at both international, national and local scale) be developed that bring benefits to African communities from conserving and sustainably managing the rainforest biome and the many ecosystem services they provide?

This short synthesis has highlighted many surprising aspects of the African rainforest biome, and how different it is in many aspects from other, perhaps better understood, rainforest regions. It has also highlighted how little we know and how much there is still to discover. There are reasons for concern, such has the heavy levels of defaunation and the potential impacts of climate change, and reasons for hope, such as the low rates of deforestation and the possible resilience of rainforests species to climate change. We call on the research and policy communities to redouble efforts to give this fascinating rainforest continent the attention it so richly deserves.

## References

[1] Malhi Y Adu-Bredu S Asare RA Lewis SL Mayaux P 2013 The past, present and future of Africa's rainforests. Phil. Trans. R. Soc. B 368, 20120293 (doi:10.1098/rstb.2012.0293) 10.1098/rstb.2012.0293 PMC3720017 23878326

[2] Mayaux P 2013 State and evolution of the African rainforests between 1990 and 2010. Phil. Trans. R. Soc. B 368, 20120300 (doi:10.1098/rstb.2012.0300) 10.1098/rstb.2012.0300 PMC3720022 23878331

[3] Rudel TK 2013 The national determinants of deforestation in sub-Saharan Africa. Phil. Trans. R. Soc. B 368, 20120405 (doi:10.1098/rstb.2012.0405) 10.1098/rstb.2012.0405 PMC3720032 23878341

[4] Mitchard ETA Flintrop CM 2013 Woody encroachment and forest degradation in sub-Saharan Africa's woodlands and savannas 1982–2006. Phil. Trans. R. Soc. B 368, 20120406 (doi:10.1098/rstb.2012.0406) 10.1098/rstb.2012.0406 PMC3720033 23878342

[5] James R Washington R Rowell DP 2013 Implications of global warming for the climate of African rainforests. Phil. Trans. R. Soc. B 368, 20120298 (doi:10.1098/rstb.2012.0298) 10.1098/rstb.2012.0298 PMC3720020 23878329

[6] Lewis SL 2013 Above-ground biomass and structure of 260 African tropical forests. Phil. Trans. R. Soc. B 368, 20120295 (doi:10.1098/rstb.2012.0295) 10.1098/rstb.2012.0295 PMC3720018 23878327

[7] Doughty CE Wolf A Malhi Y In press The legacy of the Pleistocene megafaunal extinctions on nutrient availability in the Amazon Basin. Nat. Geosci.

[8] Lewis SL 2009 Increasing carbon storage in intact African tropical forests. Nature 457, U1003 (doi:10.1038/nature07771) 10.1038/nature07771 19225523

[9] Pan YD 2011 A large and persistent carbon sink in the world's forests. Science 333, 988–993 (doi:10.1126/science.1201609) 21764754 10.1126/science.1201609

[10] Malhi Y Wright J 2004 Spatial patterns and recent trends in the climate of tropical rainforest regions. Phil. Trans. R. Soc. Lond. B 359, 311–329 (doi:10.1098/rstb.2003.1433) 15212087 10.1098/rstb.2003.1433 PMC1693325

[11] Zelazowski P Malhi Y Huntingford C Sitch S Fisher JB 2011 Changes in the potential distribution of humid tropical forests on a warmer planet. Phil. Trans. R. Soc. A 369, 137–160 (doi:10.1098/rsta.2010.0238) 21115517 10.1098/rsta.2010.0238

[12] Willis KJ Bennett KD Burrough SL Macias-Fauria M Tovar C 2013 Determining the response of African biota to climate change: using the past to model the future. Phil. Trans. R. Soc. B 368, 20120491 (doi:10.1098/rstb.2012.0491) 10.1098/rstb.2012.0491 PMC3720034 23878343

[13] Haffer J 1969 Speciation in Amazonian forest birds. Science 165, 131–137 (doi:10.1126/science.165.3889.131) 17834730 10.1126/science.165.3889.131

[14] Maslin M Malhi Y Oliver P Cowling S 2005 New views on an old forest: assessing the longevity, resilience and future of the Amazon rainforest. Trans. Inst. British Geographers 30, 477–499 (doi:10.1111/j.1475-5661.2005.00181.x)

[15] Maley J 1996 The African rain forest—main characteristics of changes in vegetation and climate from the Upper Cretaceous to the Quaternary. Proc. of the Royal Society of Edinburgh 104, 31–73 (doi:10.1017/S0269727000006114)

[16] Leal ME 2009 The past protecting the future: locating climatically stable forests in West and Central Africa. Int. J. Clim. Change Strateg. Manag. 1, 92–99 (doi:10.1108/17568690910934426)

[17] Parmentier I 2007 The odd man out? Might climate explain the lower tree alpha-diversity of African rain forests relative to Amazonian rain forests? J. Ecol. 95, 1058–1071 (doi:10.1111/j.1365-2745.2007.01273.x)

[18] Watrin J Lezine A-M Hely C 2009 Plant migration and plant communities at the time of the green Sahara. C. R. Geosci. 341, 656–670 (doi:10.1016/j.crte.2009.06.007)

[19] Oslisly R White L Bentaleb I Favier C Fontugne M Gillet JF Sebag D 2013 Climatic and cultural changes in the west Congo Basin forests over the past 5000 years. Phil. Trans. R. Soc. B 368, 20120304 (doi:10.1098/rstb.2012.0304) 10.1098/rstb.2012.0304 PMC3720025 23878334

[20] Brncic TM Willis KJ David JH Richard W 2007 Culture or climate? The relative influences of past processes on the composition of the lowland Congo rainforest. Phil. Trans. R. Soc. B 362, 229–242 (doi:10.1098/rstb.2006.1982) 17255032 10.1098/rstb.2006.1982 PMC2311427

[21] Germain B, Bernard D Joël E Emmanuel P Samuel T Sylvain B 2012 intensifying weathering and land use in iron age central Africa. Science 335, 1219–1222 (doi:10.1126/science.1215400) 22323737 10.1126/science.1215400

[22] Neumann K 2012 Comment on intensifying weathering and land use in iron age Central Africa. Science 337, 1040 (doi:10.1126/science.1221747) 22936758 10.1126/science.1221747 PMC3556809

[23] Maley J Giresse P Doumenge C Favier C 2012 Comment on intensifying weathering and land use in iron age Central Africa. Science 337, 1040 (doi:10.1126/science.1221820) 22936759 10.1126/science.1221820

[24] Gond V Fayolle A Pennec A Cornu G Mayaux P Camberlin P Doumenge C Fauvet N Gourlet-Fleury S 2013 Vegetation structure and greenness in Central Africa from Modis multi-temporal data. Phil. Trans. R. Soc. B 368, 20120309 (doi:10.1098/rstb.2012.0309) 10.1098/rstb.2012.0309 PMC3720027 23878336

[25] Hawthorne WD 1995 Ecological profiles of Ghanaian forest trees Tropical Forestry Paper 29. Oxford Forestry Institute, Department of Plant Sciences, South Parks Road, Oxford OX1 3RB, UK

[26] Barlow J Gardner TA Lees AC Parry L Peres CA 2012 How pristine are tropical forests? An ecological perspective on the pre-Columbian human footprint in Amazonia and implications for contemporary conservation. Biol. Conserv. 151, 45–49 (doi:10.1016/j.biocon.2011.10.013)

[27] McMichael CH Piperno DR Bush MB Silman MR Zimmerman AR Raczka MF Lobato LC 2012 Sparse pre-Columbian human habitation in western Amazonia. Science 336, 1429–1431 (doi:10.1126/science.1219982) 22700926 10.1126/science.1219982

[28] Nevle RJ Bird DK 2008 Effects of syn-pandemic fire reduction and reforestation in the tropical Americas on atmospheric CO_2_ during European conquest. Palaeogeogr. Palaeoclimatol. Palaeoecol. 264, 25–38 (doi:10.1016/j.palaeo.2008.03.008)

[29] Hansen MC 2008 Humid tropical forest clearing from 2000 to 2005 quantified by using multitemporal and multiresolution remotely sensed data. Proc. Natl Acad. Sci. USA 105, 9439–9444 (doi:10.1073/pnas.0804042105) 18591652 10.1073/pnas.0804042105 PMC2453739

[30] Malhi Y 2010 The carbon balance of tropical forest regions, 1990–2005. Curr. Opin. Environ. Sustain. 2, 237–244 (doi:10.1016/j.cosust.2010.08.002)

[31] FAO, JRC 2012 Global forest land-use change 1990–2050 (eds Lindquist EJ). FAO forestry paper no. 169 Rome, Italy: Food and Agriculture Organization of the United Nations and European Commission Joint Research Centre, FAO

[32] Eva HD 2012 Forest cover changes in tropical South and Central America from 1990 to 2005 and related carbon emissions and removals. Remote Sensing 4, 1369–1391 (doi:10.3390/rs4051369)

[33] Ahrends A Burgess ND Milledge SAH Bulling MT Fisher B Smart JCR Clarke GP Mhoro BE Lewis SL 2010 Predictable waves of sequential forest degradation and biodiversity loss spreading from an African city. Proc. Natl Acad. Sci. USA 107, 14 556–14 561 (doi:10.1073/pnas.0914471107) 10.1073/pnas.0914471107 PMC2930481 20679200

[34] Ernst C Mayaux P Verhegghen A Bodart C Christophe M Defourny P 2013 National forest cover change in Congo Basin: deforestation, reforestation, degradation and regeneration for the years 1990, 2000 and 2005. Glob. Change Biol. 19, 1173–1187 (doi:10.1111/gcb.12092) 10.1111/gcb.12092 23504894

[35] Gourlet-Fleury S Mortier F Fayolle A Baya F Ouédraogo D Bénédet F Picard N 2013 Tropical forest recovery from logging: a 24 year silvicultural experiment from Central Africa. Phil. Trans. R. Soc. B 368, 20120302 (doi:10.1098/rstb.2012.0302) 10.1098/rstb.2012.0302 PMC3720023 23878332

[36] Rice R Gullison R Reid J 1997 Can sustainable management save tropical forests? Sci. Am. 276, 34–39 (doi:10.1038/scientificamerican0497-44)

[37] Putz FE 2012 Sustaining conservation values in selectively logged tropical forests: the attained and the attainable. Conserv. Lett. 5, 296–303 (doi:10.1111/j.1755-263X.2012.00242.x)

[38] Nepstad DC Stickler CM Soares-Filho B Merry F 2008 Interactions among Amazon land use, forests and climate: prospects for a near-term forest tipping point. Phil. Trans. R. Soc. B 363, 1737–1746 (doi:10.1098/rstb.2007.0036) 18267897 10.1098/rstb.2007.0036 PMC2373903

[39] Bayol N 2012 Forest management and the timber sector in Central Africa. In The forests of the Congo Basin: state of the Forest 2010 (eds de Wasseige C), pp. 43–62 Luxembourg: Publications Office of the European Union

[40] Nasi R Billand A Vanvliet N 2012 Managing for timber and biodiversity in the Congo Basin. Forest Ecol. Manag. 268, 103–111 (doi:10.1016/j.foreco.2011.04.005)

[41] Doherty RM Sitch S Smith B Lewis SL Thornton PA 2010 Green future for East Africa: implications of future climate and atmospheric CO_2_ content on regional carbon cycling and biogeography. Glob. Change Biol. 16, 617–640 (doi:10.1111/j.1365-2486.2009.01997.x)

[42] Bond WJ Midgley GF 2012 Carbon dioxide and the uneasy interactions of trees and savannah grasses. Phil. Trans. R. Soc. B 367, 601–612 (doi:10.1098/rstb.2011.0182) 22232770 10.1098/rstb.2011.0182 PMC3248705

[43] Jury MR 2009 Equatorial African climate teleconnections. Theoret. Appl. Climatol. 95, 407–416 (doi:10.1007/S00704-008-0018-4)

[44] Abernethy KA Coad L Taylor G Lee ME Maisels F 2013 Extent and ecological consequences of hunting in Central African rainforests in the twenty-first century. Phil. Trans. R. Soc. B 368, 20120303 (doi:10.1098/rstb.2012.0303) 10.1098/rstb.2012.0303 PMC3720024 23878333

[45] Barnosky AD Koch PL Feranec RS Wing SL Shabel AB 2004 Assessing the causes of Late Pleistocene extinctions on the continents. Science 306, 70–75 (doi:10.1126/science.1101476) 15459379 10.1126/science.1101476

[46] Maisels F 2013 Devastating decline of forest elephants in Central Africa. PLoS ONE 8, e59469 (doi:10.1371/journal.pone.0059469) 23469289 10.1371/journal.pone.0059469 PMC3587600

[47] Walsh PD 2003 Catastrophic ape decline in western equatorial Africa. Nature 422, 611–614 (doi:10.1038/nature01566) 12679788 10.1038/nature01566

[48] Lewis SL Malhi Y Phillips OL 2004 Fingerprinting the impacts of global change on tropical forests. Phil. Trans. R. Soc. Lond. B 359, 437–462 (doi:10.1098/rstb.2003.1432) 15212095 10.1098/rstb.2003.1432 PMC1693339

[49] Fa JE Peres CA Meeuwig J 2002 Bushmeat exploitation in tropical forests: an intercontinental comparison. Conserv. Biol. 16, 232–237 (doi:10.1046/j.1523-1739.2002.00275.x) 10.1046/j.1523-1739.2002.00275.x 35701970

[50] Blake S Deem SL Mossimbo E Maisels F Walsh P 2009 Forest elephants: tree planters of the Congo. Biotropica 41, 459–468 (doi:10.1111/j.1744-7429.2009.00512.x)

[51] Effiom EO Nunez-Iturri G Smith HG Ottosson U Olsson O 2013 Bushmeat hunting changes regeneration of African rainforests. Proc. R. Soc. B 280, 20130246 (doi:10.1098/rspb.2013.0246) 10.1098/rspb.2013.0246 PMC3619513 23516245

[52] Harrison RD Tan S Plotkin JB Slik F Davies SJ Brenes-Arguedas T Itoh A Detto M 2013 Consequences of defaunation for a tropical tree community. Ecol. Lett. 16, 1–8 (doi:10.1111/ele.12102) 10.1111/ele.12102 23489437

[53] Deutsch CA Tewksbury JJ Huey RB Sheldon KS Ghalambor CK Haak DC Martin PR 2008 Impacts of climate warming on terrestrial ectotherms across latitude. Proc. Natl Acad. Sci. USA 105, 6668–6672 (doi:10.1073/pnas.0709472105) 18458348 10.1073/pnas.0709472105 PMC2373333

[54] Feeley KJ Malhi Y Zelazowski P Silman MR 2012 The relative importance of deforestation, precipitation change, and temperature sensitivity in determining the future distributions and diversity of Amazonian plant species. Glob. Change Biol. 18, 2636–2647 (doi:10.1111/j.1365-2486.2012.02719.x)

[55] Loarie SE Duffy PB Hamilton H Asner GP Field CB Ackerly DD 2009 The velocity of climate change. Nature 462, 1052–1055 20033047 10.1038/nature08649

[56] Dick CW Lewis SL Maslin M Bermingham E 2013 Neogene origins and implied warmth tolerance of Amazon tree species. Ecol. Evol. 3, 162–169 (doi:10.1002/ece3.441) 23404439 10.1002/ece3.441 PMC3568851

[57] Jury MR Matari E Matitu M 2009 Equatorial African climate teleconnections. Theor. Appl. Climatol. 95, 407–416 (doi:10.1007/s00704-008-0018-4)

[58] Washington R James R Pearce H Pokam WM Moufouma-Okia W 2013 Congo Basin rainfall climatology: can we believe the climate models? Phil. Trans. R. Soc. B 368, 20120296 (doi:10.1098/rstb.2012.0296) 10.1098/rstb.2012.0296 PMC3720019 23878328

[59] Lebel T 2011 The AMMA field campaigns: accomplishments and lessons learned. Atmos. Sci. Lett. 12, 123–128 (doi:10.1002/asl.323)

[60] Otto FEL Jones RG Halladay K Allen MR 2013 Attribution of changes in precipitation patterns in African rainforests. Phil. Trans. R. Soc. B 368, 20120299 (doi:10.1098/rstb.2012.0299) 10.1098/rstb.2012.0299 PMC3720021 23878330

[61] Asefi-Najafabady S Saatchi S 2013 Response of African humid tropical forests to recent rainfall anomalies. Phil. Trans. R. Soc. B 368, 20120306 (doi:10.1098/rstb.2012.0306) 10.1098/rstb.2012.0306 PMC3720026 23878335

[62] Shanahan TM Overpeck JT Anchukaitis KJ Beck JW Cole JE Dettman DL Peck JA Scholz CA King JW 2009 Atlantic forcing of persistent drought in West Africa. Science 324, 377–380 (doi:10.1126/science.1166352) 19372429 10.1126/science.1166352

[63] Fauset S Baker TR Lewis SL Feldpausch TR Affum-Baffoe K Foli EG Hamer KC Swaine MD 2012 Drought-induced shifts in the floristic and functional composition of tropical forests in Ghana. Ecol. Lett. 15, 1120–1129 (doi:10.1111/j.1461-0248.2012.01834.x) 22812661 10.1111/j.1461-0248.2012.01834.x

[64] Saatchi S Asefi-Najafabady S 2013 Persistent effects of a severe drought on Amazonian forest canopy. Proc. Natl Acad. Sci. USA 110, 565–570 (doi:10.1073/pnas.1204651110) 23267086 10.1073/pnas.1204651110 PMC3545782

[65] Fisher JB 2013 African tropical rainforest net carbon dioxide fluxes in the twentieth century. Phil. Trans. R. Soc. B 368, 20120376 (doi:10.1098/rstb.2012.0376) 10.1098/rstb.2012.0376 PMC3720031 23878340

[66] van der Ent RJ Savenije HHG Schaefli B Steele-Dunne SC 2010 Origin and fate of atmospheric moisture over continents. Water Resour. Res. 46 (doi:10.1029/2010WR009127)

[67] Pokam WM Djiotang LAT Mkankam FK 2012 Atmospheric water vapor transport and recycling in equatorial Central Africa through NCEP/NCAR reanalysis data. Clim. Dyn. 38, 1715–1729 (doi:10.1007/s00382-011-1242-7)

[68] Nogherotto R Coppola E Giorgi F Mariotti L 2013 Impact of Congo Basin deforestation on the African monsoon. Atmos. Sci. Lett. 14, 45–51 (doi:10.1002/asl2.416)

[69] Maniatis D Gaugris J Mollicone D Scriven J Corblin A Ndikumagenge C Aquino A Crete P Sanz-Sanchez M-J 2013 Financing and current capacity for REDD+ readiness and monitoring, measurement, reporting and verification in the Congo Basin. Phil. Trans. R. Soc. B 368, 20120310 (doi:10.1098/rstb.2012.0310) 10.1098/rstb.2012.0310 PMC3720028 23878337

[70] Asare RA Kyei A Mason JJ 2013 The community resource management area mechanism: a strategy to manage African forest resources for REDD+. Phil. Trans. R. Soc. B 368, 20120311 (doi:10.1098/rstb.2012.0311) 10.1098/rstb.2012.0311 PMC3720029 23878338

